# Productivity model and experiment of field crop spraying by plant protection unmanned aircraft

**DOI:** 10.3389/fpls.2023.1168228

**Published:** 2023-04-19

**Authors:** Weicai Qin, Panyang Chen, Baokun Wang

**Affiliations:** ^1^ Suzhou Polytechnic Institute of Agriculture, Suzhou, China; ^2^ Nanjing Institute of Agricultural Mechanization, Ministry of Agriculture and Rural Affairs, Nanjing, China; ^3^ Nanjing Institute of Technology, Nanjing, China

**Keywords:** unmanned aerial vehicle, spraying pesticide, productivity, model, evaluation method

## Abstract

Traditional agricultural production requires numerous human and material resources; however, agricultural production efficiency is low. The successful development of plant protection unmanned aerial vehicles (UAVs) has changed the operation mode of traditional agricultural production, saving human, material, and financial resources and significantly improving production efficiency. To summarize the process of improving the productivity of plant protection UAVs, this study established a productivity calculation model of UAVs based on the time composition of the UAV agricultural plant protection process, including spraying, turning, replenishment, and transfer times. The time required for the unmanned aircraft application process was counted through years of tracking the application process of eight different plant protection unmanned aircraft. Plot lengths of 100, 300, 500, 700, 1,000, 1,500, 2,000, 2,500, 3,000, and 3,500 m were established to calculate the theoretical productivity. The results showed that the productivity of different types of plant protection UAVs increased with an increase in plot length in the range of 100 to 1,500 m; however, when the plot length reached a certain value, the productivity growth rate slowed down or even decreased slightly. Simultaneously, based on the working area per 10,000 mu, the recommended plot length and the number of configured models for different models were recommended. If the plant protection UAV was distinguished by electric and oil power, the time utilization rate of electric plant protection UAVs was 72.7%, and the labor productivity was 56.4 mu/person·h. In contrast, the time utilization rate of the heavy load oil-powered plant protection unmanned aircraft was 86%, and the labor productivity was 63.5 mu/person ·h. This study can support plant protection UAV enterprises to optimize equipment efficiency, provide evaluation methods for the operation efficiency assessment of plant protection UAVs, provide a reference for the selection of plant protection UAVs, and provide a basis for field planning.

## 1 Introduction

The plant protection unmanned aerial vehicle (UAV) has several advantages compared with the ground boom sprayer, which include high operation efficiency, strong ability to handle emergencies, low operating cost, and labor intensity (Lou et al., 2017). Specifically, it solves the difficult problem of ground equipment and manual work in the late stage of crop growth. Furthermore, with the further implementation of China’s land transfer policy, the scale of agricultural production has continued to expand (Xue and Lan, 2013; Feng and Yang, 2014), the cost of rural labor has continued to rise, and the demand for unmanned aerial vehicle application in agricultural plant protection has become increasingly strong (Kirk, 2003). Therefore, technical research, equipment development, and operation mode formulation of plant protection UAV spraying operations have become the research hotspots of scientific research units, enterprises, and promotion departments in plant protection machinery.

Recently, studies have reported considerable innovations in improving the quality and efficiency of plant protection unmanned aerial vehicle operations from spraying operation parameter matching, equipment performance optimization, and operation path planning (Zhang et al., 2021). Several studies have been conducted on the application of plant protection UAVs, such as operating speed (Kirk et al., 2001; Qin et al., 2014), spray volume and height (Lan et al., 2010), spray width (Zhu et al., 2019), droplet size (Qiu et al., 2013), natural wind speed (Chen et al., 2017a), and airflow field (Carlton, 1999; Chen et al., 2017b), on the influence of droplet deposition, establishing an advanced and specific spraying operation parameter model and its evaluation method (Zhang et al., 2015; Wang et al., 2016a).

Productivity improvement studies mainly focused on the path planning of pesticide application (Wang et al., 2016b), such as the accuracy and control of the route of plant protection unmanned aircraft based on the farmland environment (Ru et al., 2012; Yang et al., 2017), the development of an efficient matching algorithm for plant protection unmanned aircraft operations (Xu et al., 2017), the deployment and decision-making of the UAV flying defense team (Cao et al., 2019), and the monitoring technology of the pesticide application state of the plant protection unmanned aircraft (Xu et al., 2017; Yang et al., 2019).

Current studies mainly focus on improving the operation quality of plant protection UAVs and the reliability of spraying equipment, which provides much technical support for popularizing and applying plant protection UAVs. The productivity of plant protection UAVs is very important for farmland size planning and equipment configuration for intensive production; however, there are no relevant studies. Therefore, this study collected production data of plant protection UAVs from 2016 to 2020 to summarize the method of enhancing their production process. Furthermore, the study proposes the appropriate plot length and configuration quantity for each model under stable labor productivity, providing data support for applying plant protection UAVs.

## 2 Material and methods

### 2.1 Technical index of productivity

The technical indicators of plant protection unmanned aerial vehicle spraying operation productivity include hourly working time productivity, net spraying hourly productivity, shift time utilization, and labor productivity. Equations 1–4 (Qiao et al., 2016) are as follows:


(1) $$\begin{array}{c} W_{b}=\frac{U}{T_{T}} \end{array}$$

(2) $$\begin{array}{c} W_{s}=\frac{U}{T_{z}} \end{array}$$

(3) $$\begin{array}{c} \tau =\frac{T_{z}}{T_{T}}\cdot 100\% \end{array}$$

(4) $$\begin{array}{c} G_{j}=\frac{W_{b}}{A_{j}} \end{array}$$

where *W_b_* is the actual hourly productivity, mu/h; *U* is the actual working area, mu; *T_T_* is the flight time, h; *W_S_* is the hourly productivity of net spraying, mu/h; *T_Z_* is the net spraying time, h; *τ* is the time utilization, %; *G_j_* is labor productivity, mu/person·h; and *A_j_* is the number of crew members, people.

### 2.2 Job productivity model

#### 2.2.1 Model basis

The productivity of plant protection unmanned aircraft was acquired according to GB/5667-2008 (Standardization Administration of China, 2008):


(5) $$\begin{array}{c} W=0.36B\cdot v\cdot \tau \end{array}$$

where *W* is the productivity, mu/h; *B* is the spraying amplitude of plant protection unmanned aircraft, m; *v* is the application speed, m/s; and *τ* is the time utilization rate, the ratio between the net application time and the total time during the application duration of plant protection UAV. Plant protection UAV pesticide application requires frequent take-off and landing, and the time utilization model is shown in Equation 6.


(6) $$\begin{array}{c} \tau =\frac{\sum^{}T_{zi}}{\sum T_{Ti}} \end{array}$$

where *T_zi_* is the net spraying time when spraying the *i*th tank of spray liquid, s, and *T_Ti_* is the total time for spraying the *i*th tank of spray liquid, s.

#### 2.2.2 Total operation time model

The reliability of the plant protection UAV is not part of the content of this test, the scale of the test is large, and communication and coordination will cause time delays. Therefore, the total time of this test is the sum of the time of each operation sortie statistical spraying task. The spraying operation of the plant protection UAV includes preparation before the first operation (assembly, maintenance, and debugging), supply (maintenance, dosing, refueling, or battery replacement), attitude adjustment before the operation (stabilizing the rotor speed and entering the flight trajectory), spraying, U-turn (acceleration and deceleration and U-turn), return and flameout (empty travel and rotor stop), and supply point transfer and other links. The total time model of its work is shown in Equation 7:


(7) $$\begin{array}{c} T_{Ti}=T_{fp}+\sum^{}\left(T_{si}+T_{ami}+T_{ai}+T_{ri}+T_{toi}+T_{sui}\right) \end{array}$$

where *T_fp_* is the preparation time before the first operation, s; *T_si_* is the total refill time at the *i*th tank of solution, s, including refilling, refueling, battery replacement, maintenance, and other time; *T_ami_* is the adjustment time before the application operation at the *i*th tank of liquid, s, time from ignition to when spraying begins; *T_ai_* is the net spray time when the *i*th tank is sprayed, s; *T_ri_* is the turnaround time when the *i*th tank of medicine, s; *T_toi_* is the return flameout time at the *i*th tank of medicine, s, from the end of spraying until the rotor stops rotating; and *T_sui_* is the transfer time of the *i*th resupply point, s.

Research on the time composition and representation of plant protection unmanned aircraft production operations aims to improve the basic theory of production performance and testing methods, summarize the production performance testing methods of agricultural machinery, construct an unmanned aircraft production efficiency model, and propose processing solutions to improve the production performance of different unmanned aircraft platforms in response to their production data.

#### 2.2.3 Replenishment time calculation model

The net spraying time is mainly affected by the maximum travel, replenishment times, and time the plant protection unmanned aircraft can operate with a full tank of spray liquid. Among them, the maximum stroke that can be operated with a full tank of spray liquid is related to the capacity of the tank, the operating speed, and the total flow of the nozzle. The model calculation is shown in Equation 8:


(8) $$\begin{array}{c} L_{max}=\frac{60Q\cdot v}{q} \end{array}$$

where *L_max_* is the maximum stroke that can be operated with a full tank of liquid, m; *Q* is the (maximum) spray liquid loading capacity, L; and *q* is the total flow rate of the nozzle, L/min. The flight path is planned with the fewest supply points and the fewest number of U-turns during spraying operations. For example, suppose the length of the plot is *L*, and the width of the plot is *B_f_*; the number of operational trips of the plant protection UAVs to complete the application of a field is the ratio of the width of the field to the width of the spray operation, and combined, thus, the calculation is shown in Equation 9:


(9) $$\begin{array}{c} N_{x}=\left[\frac{B}{B_{f}}\right] \end{array}$$

where *B* is the spraying working width, m; *B_f_* is the width of the plot, m; and *N_x_* is the number of operating strokes. The width of the plot should be designed as much as possible to be double the spray width of the plant protection unmanned aircraft to ensure that the spraying operation does not occur with heavy and missing spraying and spreading of empty strokes.

1) When $\;L_{max}\leq 2L$ , the replenishment points are


(10) $$N_{b}=\left[\frac{2L}{L_{max}}\right]$$

The number of refills is $N_{s}=N_{b}\cdot \left[\frac{N_{x}}{2}\right]+\left[\frac{N_{x}\left[2L-\left(N_{b}-1\right)L_{max}\right]}{2L_{max}}\right].$

2) When $\;L_{max}>2L$ , only one resupply point is set up on one side of the ground.

The number of refills is


(11) $$N_{s}=\left[\frac{N_{x}}{\left[\frac{L_{max}}{2L}\right]}\right]$$

#### 2.2.4 Productivity calculation model

To obtain the productivity model Equation 12 of plant protection unmanned aerial vehicle spraying operation, substitute Equations 6–11 into Equation 5:


(12) $$\begin{array}{c} W=0.36Bv\frac{\sum_{i=1}^{N_{a}}T_{ai}}{T_{fp}+\sum_{i=1}^{N_{a}}\left(T_{si}+T_{ami}+T_{ai}+T_{toi}\right)+\sum_{i=1}^{N_{s}}T_{ri}+\sum_{i=1}^{N_{b}}T_{sui}} \end{array}$$

where *N_s_* is the number of refills, *N_am_* is the number of adjustments before application operations, *N_to_* is the number of returns and flameouts and *N_a_* is the number of spraying operations frame. *N_s_* = *N_am_* = *N_to_* = *N_a_*.The number of turns is *N_r_* = *N_b_* (*N_x_* − 1). Test statistics determine each time item in the formula, and the time data of each model are shown in **Table 1**.

**Table 1:** 1 Time composition test data for each unit.

Model	Preparation time before application *T_fp_*/s	Replenishment time *T_s_*/s	Adjust time before spraying operation *T_am_*/s	Net spray time *T_a_*/s	Turnaround time *T_r_*/s	Return, turn off time *T_to_*/s	Supply point transfer time *T_su_*/s
CE20	325	256	40	750	3	36	1,200
P20	350	345	30	600	2	32	1,200
AT-30	900	240	105	1,500	10	75	1,200
4DE1000	185	305	45	460	6.5	25	1,200
MG-1S	450	350	10	400	2	20	1,200
HY-B-16L	250	305	30	400	6	45	1,200
3WQF120-12	358	180	42	500	3	51	1,200
LF-D10	485	285	31	250	3.8	30	1,200

### 2.3 Plant protection UAV

**Figure 1:**
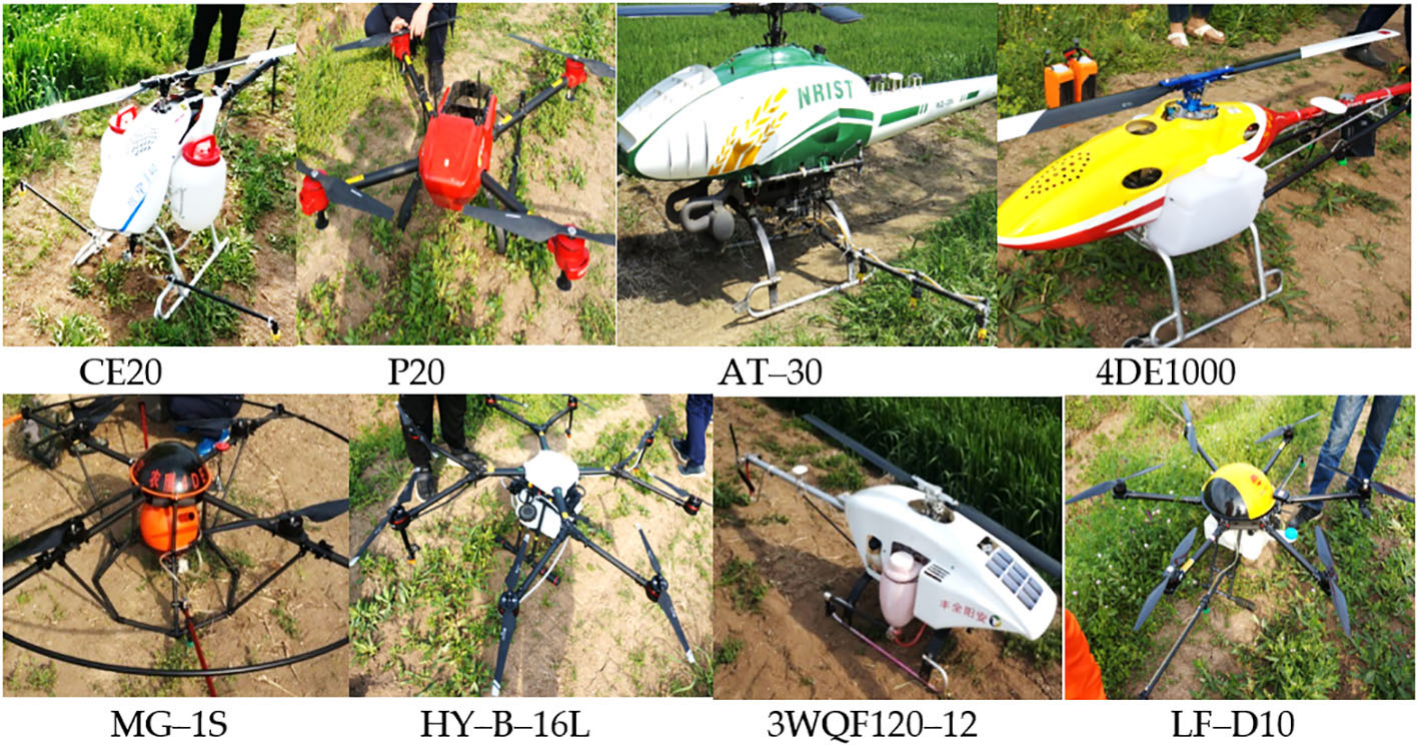
The UAVs used in testing. Note: the P20 is installed with four nozzles, but only two nozzles spray at a time. UAVs, unmanned aerial vehicles.

A total of eight types of plant protection unmanned aircraft with different power, atomization methods, and the number of rotors were selected for this test. As shown in **Figure 1**. The power included electric and oil power, and the rotors included single, four, six, and eight rotors. The weight was 8–30 kg. The basic parameters of the test plant protection UAVs are shown in **Table 2**.

**Table 2:** Basic parameters of plant protection UAV participating in the test.

Model	Power type	Sprinkler type	Number of nozzles/piece	Nozzle flow/L/min	Tank capacity Q/L	Amplitude/m	Velocity/m/s	Single operation time/min
CE20	Electric single rotor	Hydraulic atomization	2	0.8	20	6	5	20
P20	Electric quadrotor	Centrifugal atomization	4 (2)	0.4	8	3	4	25
AT-30	Oil powered single rotor	Hydraulic atomization	6	0.25	30	8	5	20
4DE1000	Electric quadrotor	Hydraulic atomization	4	0.33	10	4.5	6	10
MG-1S	Electric octa-rotor	Hydraulic atomization	4	0.38	10	4	4	22
HY-B-16L	Electric single rotor	Hydraulic atomization	5	0.48	16	7	5	33
3WQF120-12	Oil powered single rotor	Hydraulic atomization	2	0.73	12	4	5	25
LF-D10	Electric hexacopter	Hydraulic atomization	4	0.6	10	4	4	15

UAVs, unmanned aerial vehicles.

### 2.4 Test conditions and methods

Eight adjacent plots were selected for allocation to each plant protection UAV, each 730 m in length, with CE20, P20, 4DE1000, HY-B-16L, MG-1S, LF-D10, 3WQF120-12, and AT-30 operating plots of 60.3, 51.7, 21.5, 21.5, 21.1, 19.1, 41.8, and 52.8 mu, respectively.

Based on continuous tracking of plant protection unmanned aircraft productivity tests for many years, Excel 2010 software was used to process data and draw curves, among which the key replenishment point calculation condition statement function was as follows:

IF(N_b_ = 1,CEILING(20/CEILING(L_max_/(2 * L),1),1),((N_b_ − 1) * 10 + CEILING(20 *(2 * L − (N_b_ − 1) * L_max_)/(2 * L_max_),1))).

The test crews were professionals from each plant protection unmanned aircraft company, and each crew developed his/her operation plan according to the characteristics of his/her unmanned aircraft. In addition, each crew had a researcher responsible for recording the total effective operation time, the net spraying time, and the time consumption of each time item.

### 2.5 Data acquisition and recording

The total time consumption and net spraying time of the three plant protection operations were recorded and averaged as shown in **Table 3**. In addition, the technical indicators such as time utilization, hourly productivity, net spraying hourly productivity, and labor productivity were calculated according to Equations 1–4, respectively, according to the number of crew members and the actual operating area of each model. The calculation results are shown in **Table 3**.

**Table 3:** Productivity technical indicators of different types of plant protection UAVs.

Technical indicators	CE20	P20	4DE1000	HY-B-16L	MG-1S	LF-D10	3WQF120-12	AT-30
Working area/mu	60.300	51.700	21.500	21.500	21.100	19.100	41.800	52.800
Total time/h	2.000	1.900	1.030	0.880	1.100	1.100	2.500	2.000
Number of crew members/person	3	2	2	3	2	2	3	4
Net spray time/h	0.9	0.85	0.34	0.36	0.4	0.45	0.5	0.45
Time utilization/%	45	44.7	33	40.9	36.4	40.9	20	22.5
Actual hourly productivity, mu/h	30.150	27.211	20.874	24.432	19.182	17.364	16.720	26.400
Net spray hour productivity, mu/h	67.000	60.824	63.235	59.722	52.750	42.444	83.600	117.333
Labor productivity, mu/person·h	10.050	13.605	10.437	8.144	9.591	8.682	5.573	6.600

UAVs, unmanned aerial vehicles.

The productivity test was conducted together with the efficacy test to facilitate the organization of the test. A fixed operating area was arranged for different units. So that the normal application operations are not affected, each operational sortie was used as a statistical unit to track and test various time items occurring during application operations of different models and to record the time consumed by each time item. Therefore, the data in **Table 1** are a combination of years of test data for each time item with abnormal data exclusion, and the average of the time items of all sorties during the operation period is the test result, as shown in **Table 1**. The transfer time of the resupply point was set to 20 min because the crews of different types of plant protection UAVs were configured by the ability of one crew to complete the transfer at one time, and the article was calculated according to the transfer length of 600 m and the weight walking speed of 0.5 m/s.

## 3 Results and discussion

### 3.1 Comparative analysis of technical indicators of productivity of different models

As observed from Equation 2, the net spraying hourly productivity of plant protection UAVs is an important indicator of the inherent performance of plant protection unmanned aircraft, including spraying width, operating speed, and tank capacity. The actual hourly productivity of the impact indicators, in addition to the inherent performance of the equipment and time utilization, is also a key factor affecting the hourly productivity.

The test results in **Table 3** completely verified the law, such as the AT30 plant protection UAV with a tank capacity of 30 L, a spraying width of 8 m, and an operating speed of 5 m/s. Therefore, the net spraying time of this model was the highest among all models, reaching 117.3 mu/h. In contrast, the LF-D10 plant protection UAV has a tank capacity of 10 L, a spraying width of 4 m, and an operating speed of 4 m/s; thus, the net spraying time of this model was the lowest among all models, only 42.4 mu/h. However, the complexity of the operations and the level of organizational proficiency of the different models lead to significant differences in time utilization, ranging from a minimum of 20% to a maximum of 45%, which was affected by time utilization AT30’s actual hourly productivity of 26.4 mu/h, which was lower than CE20’s actual hourly productivity of 30.15 mu/h. Furthermore, the labor productivity calculation model Equation 4 shows that the labor productivity is related to the actual hourly productivity and unit configuration labor; therefore, the labor productivity of P20 with a 2-labor unit configuration can reach up to 13.6 mu/person·h. In contrast, the actual labor productivity of AT30 was only 6.6 mu/person·h owing to the low time utilization and a large amount of labor allocated to the unit.

From the above analysis, the actual operational efficiency of plant protection unmanned aircraft application is affected by the inherent performance of the unit as well as closely related to the reasonable configuration of the unit’s labor force, the level of organization and coordination of the crew, and the degree of operational proficiency (time utilization).

### 3.2 Analysis of the relationship between theoretical productivity and plot length

**Figure 2:**
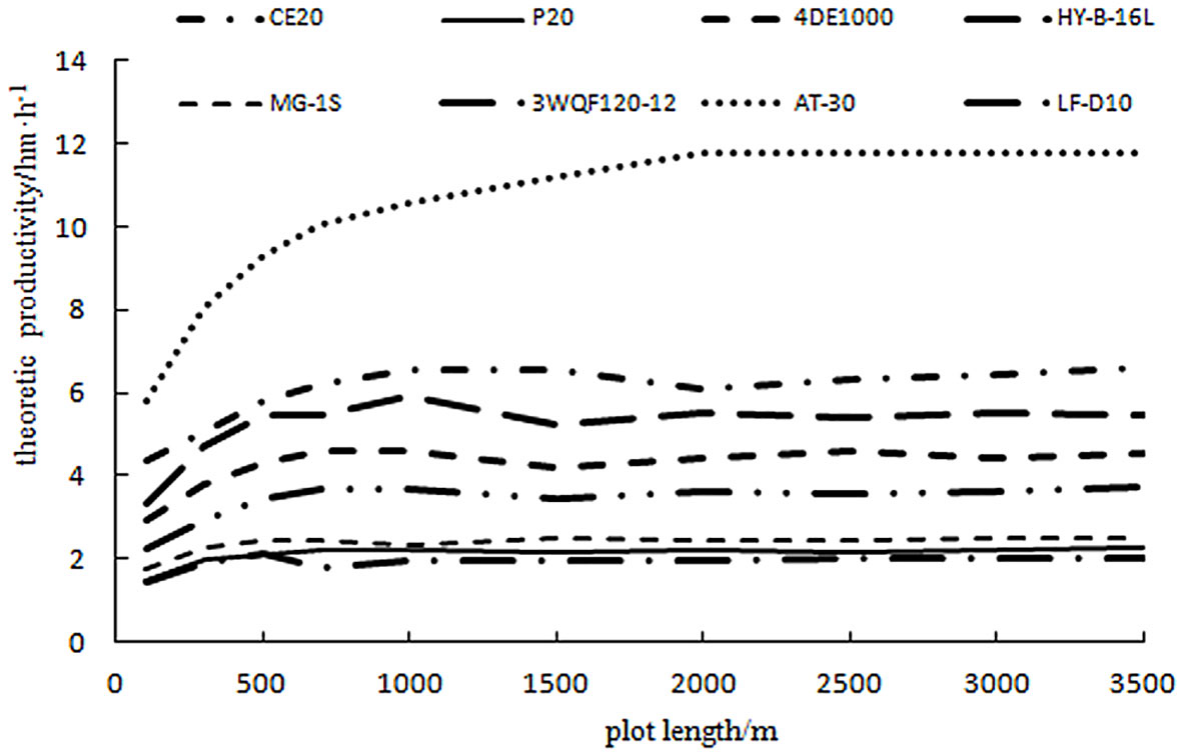
Curves of theoretical productivities at different plot lengths.

From the time consumption data of the plant protection unmanned aircraft in **Table 1**, the variation law of crew productivity and plot conditions can be derived according to the productivity model Equation 12. To study the effect of plot length on the productivity of each plant protection UAV, the width of the operating plot for each model in the study was calculated according to 20 operating strokes, and the plot lengths were calculated from 100, 300, 500, 700, 1,000, 1,500, 2,000, 2,500, 3,000, and 3,500 m. Combined with the data in **Table 1** using Excel 2010 software to prepare the calculation program, the theoretical productivity of each model with the plot length variation law is shown in **Figure 2**.

Analysis of **Figure 2** shows that the productivity of different models of plant protection UAVs increases with plot length on the 100–1,500-m interval, but the increase varies. When the block length reaches a certain value, the rate of increase in productivity slows down or even decreases slightly.

To derive the plot lengths corresponding to the maximum productivity of the different models, the curves in **Figure 2** focused on the 100–1,500-m plot length interval to obtain the optimum plot lengths for the different models and their corresponding theoretical productivity, as shown in **Table 4**. Analysis of the data in **Table 4** shows that the theoretical productivity of the plant protection unmanned aircraft can reach a maximum of 10.1 ha/h. The theoretical productivity of each model also varies greatly because the theoretical productivity is related to the time of each statistical item in **Table 1** and is more affected by key parameters such as spraying width, operating speed, and the tank capacity of the plant protection unmanned aircraft. However, theoretical productivity does not consider the differences in organizational coordination, staffing, and requirements of each model, and these parameters are important indicators of time utilization and labor productivity.

**Table 4:** Suitable plot lengths and the number of configurations for each model.

Model	Plot length/m	Theoretical productivity ha/h	Configuration quantity/rack˙10,000 mu^−1^
CE20	1,000–1,500	6.5	4
P20	700–1,000	2.2	9
AT-30	2,000–2,500	10.1	3
4DE1000	700–1,000	4.58	5
MG-1S	500–700	2.43	8
HY-B-16L	700–1,000	5.9	4
3WQF120-12	700–1,000	3.8	6
LF-D10	300–500	2.11	10

According to the operating area of 10,000 mu, the effective period of each pest control is calculated by 5 days, and each unit works 8 h/day; combined with the maintenance requirements of each model, **Table 4** recommends the number of plant protection unmanned aircraft required for the operating area of 10,000 mu for different models.

### 3.3 Labor productivity improvement

From the model Equation 5, the productivity of plant protection UAVs is related to the spraying width, operating speed, and time utilization; the optimal spraying width and operating speed are determined for a specific plant protection UAV type to improve productivity only by the time utilization of the application operation. Model Equation 4 reveals that labor productivity is related to productivity and has an important relationship with the number of units configured; therefore, reducing the number of units is the most direct way to improve labor productivity.

**Figure 3:**
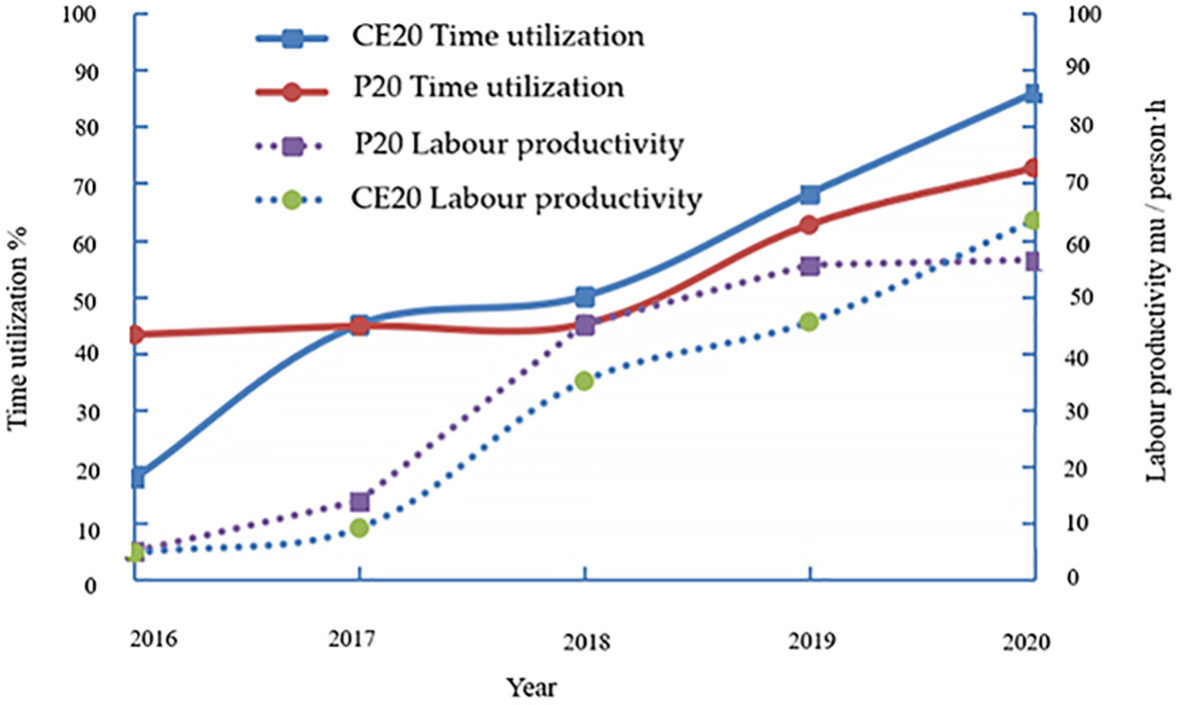
Production efficiency improvement.

As observed in **Figure 3**, with the advancement of the battery technology of electric UAVs and the reliability of oil-powered plant protection UAVs, the time utilization rate of electric plant protection UAVs increased from 43% in 2016 to 72.7% in 2020, and the labor productivity increased from 4.85 mu/person·h in 2016 to 56.4 mu/person·h in 2020. In contrast, the time utilization rate of oil-powered aircraft increased from 17.8% in 2016 to 86% in 2020, and labor productivity increased from 4.55 mu/person·h in 2016 to 63.5 mu/person·h in 2020. The reason for this is the high failure rate of the early oil-powered plant protection unmanned aircraft. The complex take-off and landing process leads to low efficiency, and with the reliability of the aircraft to improve the equipment control, and autonomous improvement, efficiency greatly improved.

## 4 Conclusion

1) This study determined the time composition of plant protection UAV application operations and established a mathematical model of the variation pattern of operational productivity and plot length.
2) The actual operational efficiency of plant protection UAV application is affected by the inherent performance of the crew and is closely related to the reasonable configuration of the crew’s labor force, the level of organization and coordination of the crew, and the degree of operational proficiency (time utilization).
3) The optimal plot lengths of different types of plant protection UAVs and their corresponding theoretical productivity were obtained, and the number of UAVs required for each 10,000 mu of operating area was recommended.
4) As battery technology for electric UAVs and the reliability of oil-powered plant protection UAVs progressed, the time utilization of electric and oil-powered plant protection UAVs increased from 2016 to 2020.

The study conclusions obtained can support plant protection UAV enterprises to optimize equipment efficiency, provide evaluation methods for the operational efficiency assessment of plant protection UAVs, provide a reference for the selection of plant protection UAVs, and provide a basis for field planning.

## Data availability statement

The original contributions presented in the study are included in the article/supplementary material. Further inquiries can be directed to the corresponding author.

## Author contributions

WQ designed the study. WQ, PC, and BW performed most experiments. WQ analyzed the data and wrote the first draft of the manuscript. PC revised the manuscript. BW supervised the project and reviewed the manuscript. All authors contributed to the article and approved the submitted version.
